# Endoscopic Submucosal Dissection for Treatment of Early-Stage Cancer or Precancerous Lesion in the Upper Gastrointestinal Tract in Patients with Liver Cirrhosis

**DOI:** 10.3390/jcm12206509

**Published:** 2023-10-13

**Authors:** Yuyong Tan, Yumin Qing, Deliang Liu, Jian Gong

**Affiliations:** 1 Department of Gastroenterology, The Second Xiangya Hospital, Central South University, Changsha 410011, China; tanyuyong@csu.edu.cn (Y.T.); qingyumin@csu.edu.cn (Y.Q.); deliangliu@csu.edu.cn (D.L.); 2 Research Center of Digestive Disease, Central South University, Changsha 410011, China; 3 Clinical Research Center for Digestive Disease in Hunan Province, Changsha 410011, China

**Keywords:** early-stage cancer, precancerous lesion, endoscopic submucosal dissection, liver cirrhosis

## Abstract

(1) Background: Endoscopic submucosal dissection (ESD) has been widely accepted as the standard method for treating early-stage cancer or precancerous lesions in the upper gastrointestinal tract; however, it may be difficult in patients with liver cirrhosis due to clinical challenges such as coagulation dysfunction, presence of gastroesophageal varices, etc. We aimed to demonstrate the safety and efficacy of ESD in these populations. (2) Methods: The clinical data of patients were retrospectively collected and analyzed. Inclusion criteria of the study were: a. patients with liver cirrhosis; b. patients who underwent ESD; c. patients who were diagnosed with early-stage cancer or precancerous lesions in the upper gastrointestinal tract. (3) Results: Eight patients were enrolled from April 2019 to April 2023, of whom three were male and five were female, with ages ranging from 43 to 70 years old. Seven lesions were located in the stomach and one other lesion was in the esophagus. ESD was performed successfully in all eight patients, and the resected lesion size ranged from 2 to 6 cm. Only one patient encountered postoperative complications, namely, chest pain and fever. No recurrence was noticed during a follow-up of 3 to 45 months. (4) Conclusions: ESD may serve as a safe and effective method for treating upper gastrointestinal early-stage cancer or precancerous lesions in patients with liver cirrhosis.

## 1. Introduction

Esophageal and gastric cancers, which we call upper gastrointestinal cancers, are two of the most common cancers around the world with high incidence (ranking eighth and fifth among all cancers, respectively) and mortality (ranking sixth and fourth among all cancers, respectively) rates, especially in Asian countries such as China, Korea, and Japan [1]. The overall 5-year survival rates of advanced esophageal and gastric cancer are lower than 30% even after comprehensive therapy such as surgery, chemotherapy, immunotherapy, etc., while the rates exceed 90% when found at an early stage and treated timely [2,3]. With the development of patients’ awareness and equipment of cancer screening, more and more early-stage cancer and precancerous lesions are found, which has ultimately declined cancer-related mortality [4]. The treatment of early-stage cancer or precancerous lesions mainly includes endoscopic and surgical resection; endoscopic resection is less expensive, less traumatic, and is associated with faster recovery and a better quality of life compared to surgery, without any compromise in overall survival and disease-specific survival [5,6,7,8]. Among various endoscopic methods, endoscopic submucosal dissection (ESD) is superior to others, with a higher en bloc resection and curative resection rate [9,10]; thus, ESD has been widely accepted as the primary treatment option for early esophageal and gastric cancers in many countries.

Liver cirrhosis is another prominent health problem in Asian countries, especially in China, given the high incidence of hepatitis B virus (HBV) and hepatitis C virus (HCV) infection [11]. Patients with liver cirrhosis are considered as high-risk candidates for invasive treatments including ESD due to their tendency to bleed, the vulnerability of tissues, the increased rate of postoperative infection, poor tolerance to anesthesia, etc. [12]. Although several studies have demonstrated that endoscopic treatments including ESD are safe and effective in this population [13,14,15,16,17,18,19,20], many concerns have not been solved satisfactorily or sometimes their conclusion was contradictory. Kim et al. [14] found that liver cirrhosis was associated with a higher rate of cancer recurrence, while Choe et al. [13] failed to demonstrate the difference regarding cancer recurrence rate, but they found the overall mortality rate was higher in the liver cirrhosis group. Two recent studies have demonstrated that the presence of gastroesophageal varices (GOV) does not compromise the safety and efficacy of endoscopic resection [17,21]; however, the management of coexisting GOV remains debatable. What is more, no consensus was reached on many other concerns such as proper perioperative management, the minimum requirement of platelet (PLT) count, etc. In the present study, we retrospectively collected and analyzed the clinical data of patients with coexisting early esophageal/gastric cancers or precancerous lesions and liver cirrhosis, aiming to explore the safety and efficacy of ESD for early-stage upper gastrointestinal cancers or precancerous lesions in liver cirrhosis patients, as well as to explore the proper perioperative management.

## 2. Materials and Methods

### 2.1. Patients

We retrospectively collected the clinical data of enrolled patients at the Department of Gastroenterology, The Second Xiangya Hospital of Central South University, from April 2019 to April 2023. Patients who met the following criteria were enrolled in the study: (1) patients diagnosed with early-stage cancers or precancerous lesions in the upper gastrointestinal tract; (2) patients diagnosed with liver cirrhosis either before or simultaneously with the discovery of the cancerous or precancerous lesion; (3) patients who underwent ESD at our hospital. The exclusion criteria of the study were as follows: (1) patients with incomplete information; (2) as we evaluate the safety and efficacy of ESD in these patients, patients who underwent surgery for the cancerous or precancerous lesion who were not enrolled; (3) patients who underwent ESD at other hospitals but sent the resected specimen to our hospital for confirmed diagnosis were also excluded due to the difficulty in calculating the exact liver cirrhosis incidence among patients with upper gastrointestinal cancerous or precancerous lesions; (4) patients with severe cardiopulmonary disease who could not tolerate the ESD procedure; (5) ESD was not encouraged in patients with a short life expectancy due to severe liver (or other organ) dysfunction due to liver cirrhosis. This study was conducted in accordance with the Declaration of Helsinki and was approved by the ethics committee of our hospital. All patients were informed of the potential risks of ESD and alternative treatment methods and signed the informed consent form.

### 2.2. Endoscopic Equipment and Accessories

The ESD procedures were performed under general anesthesia with endotracheal intubation. A single-channel endoscope (GIF-Q260J; Olympus Corp., Tokyo, Japan) was used, with a transparent cap (D-201-11802, Olympus) attached to the front. A carbon dioxide insufflator (UCR; Olympus) was used. Other equipment and accessories used during the ESD procedure included a high-frequency generator (ICC 200; ERBE Elektromedizin GmbH, Tübingen, Germany), a dual knife (KD-650 L/Q, Olympus Corp., Tokyo, Japan), an argon plasma coagulation unit (APC300; ERBE), and an injection needle (NM-4L-1; Olympus). A solution consisting of 100 mL saline + 5 mL 0.2% indigo carmine + 1 mg epinephrine was used for submucosal injection during the ESD procedure.

### 2.3. ESD Procedure

**Figure 1:**
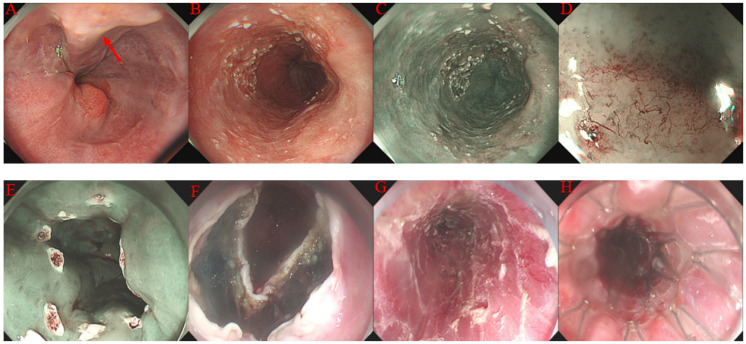
Case illustration of endoscopic submucosal dissection (ESD) for superficial esophageal cancer. (**A**) endoscopic image showing esophageal varices (red arrow) at the lower esophagus. (**B**) the endoscopic image showing a 0-IIb lesion at the lower esophagus. (**C**) narrow band image of the lesion. (**D**) narrow band image and magnifying endoscopy revealing intraepithelial papillary capillary loop of type B1 and B2. (**E**) marking the margin of the lesion. (**F**) mucosal and submucosal excision at the anal side of the lesion. (**G**) the wound surface after ESD. (**H**) a fully covered metal stent was inserted after completion of ESD.

**Figure 2:**
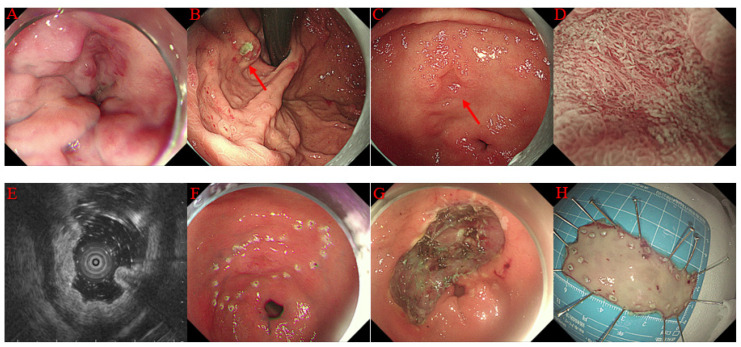
Case illustration of endoscopic submucosal dissection (ESD) for early gastric cancer. (**A**) endoscopic image showing esophageal varices. (**B**) endoscopic image showing gastric fundus ulceration owing to endoscopic cyanoacrylate injection for gastric varices (red arrow). (**C**) a 0-IIa + IIc lesion (red arrow) at the lesser curvature of the gastric antrum. (**D**) narrow band image and magnifying endoscopy revealed that the lesion had a demarcation line and irregular microsurface pattern. (**E**) endoscopic ultrasonography revealed that the lesion was restricted to the mucosal layer. (**F**) marking the margin of the lesion. (**G**) the wound surface after ESD. (**H**) the resected specimen.

The ESD was performed as previously reported [22,23]. Briefly, the procedure had the following steps: (a) narrow band imaging and magnifying endoscopy were used to determine the area and boundary of the lesions; (b) marking the margin of the lesion, usually 3 to 5 mm beside the boundary of the lesion; (c) submucosal injection to provide a submucosal cushion to facilitate dissection; (d) for gastric lesions, usually circumferential mucosal incision was performed first, while only partial mucosal incision was performed initially for esophageal lesions, and the remaining mucosal incision was performed during the ESD procedure; (e) dissection of the lesion, submucosal injection was repeated during the ESD procedure when necessary; (f) management of the wound surface. Visible vessels were prophylactically managed with electrocoagulation or hemostatic forceps. Endoclips closure or stent (for esophageal lesion only) was used for patients with an unintentional muscularis propria layer injury or with potential risk of perforation. Figure 1 and Figure 2 depict an example of esophageal and gastric ESD.

### 2.4. Preoperative and Postoperative Management

Platelet was infused preoperatively in patients with a platelet count of less than 50 × 10^9^/L; fresh frozen plasma was infused for patients with coagulation dysfunction (mainly impaired INR), and albumin was infused for patients with blood albumin <30 g/L. Intraoperative bleeding was recorded as the necessity of endoscopic hemostasis during ESD [16]. Postoperative bleeding was defined as any of the following: apparent hematemesis or melena, unstable vital signs, or a >2 g/dL decrease in hemoglobin concentration after ESD [21]. Patients were kept nil per os for 1 to 2 days, then on a liquid diet for 3 days, and gradually switched to a normal diet within 2 weeks. Proton pump inhibitor was given intravenously during the hospital stay and then orally for another 4 weeks for gastric lesions. Postoperative observations included vital signs, recording of complaints of abdominal/ chest pain, dyspnea, hematemesis, melena, and abdominal distention, and an abdominal/chest examination.

### 2.5. Pathological Assessment

The resected specimens were fixed, embedded in paraffin, sent to the Department of Pathology, and then sectioned. Hematoxylin and eosin staining, or sometimes immunohistochemical staining was performed. En bloc resection is defined as a resection that results in the removal of a single piece of tissue. R0 resection refers to histologically complete tumor removal with tumor-free lateral and basal margins, while curative resection is defined as resected neoplasia restricted to the epithelium (m1) or lamina propria (m2) but not involving the muscularis mucosa (m3), with neoplasia-free vertical and radial margins and no lymphatic or vascular invasion.

### 2.6. Pathological Assessment

Surveillance endoscopy was performed at 3, 6, and 12 months and annually thereafter to observe the healing of the wound and check for any signs of complications (such as stricture for esophageal lesion) and recurrence.

### 2.7. Statistical Analysis

Only descriptive analysis was performed due to the small sample size. Continuous variables are presented as specific values for each patient, and categorical variables are presented as frequency with percentage.

## 3. Results

### 3.1. Basic Clinical Characteristics

From April 2019 to April 2023, a total of 106 patients with early esophageal cancers or precancerous lesions and 342 patients with early gastric cancers or precancerous lesions received ESD treatment in our hospital; among whom, eight patients had comorbid liver cirrhosis and seven of them had early-stage cancer or precancerous lesions in the stomach, while the other one had a lesion in the esophagus. Thus, the incidence of comorbid liver cirrhosis was 0.94% (1/106, for early esophageal cancers or precancerous lesion) and 2.05% (7/342, for early gastric cancers or precancerous lesion) in our hospital.

Of the eight patients, three were male and five were female, with ages ranging from 43 to 70 years old. The course of liver cirrhosis was 0 to 5 years (0 means that liver cirrhosis and the upper gastrointestinal lesion were found simultaneously), and four patients had GOV and two of them had a history of endoscopic variceal therapy. Thrombocytopenia, anemia, impaired INR, and hypoalbuminemia were found in six, six, three, and six patients, respectively. Five patients were classified as Child–Pugh level A, while the other three were classified as Child–Pugh level B (see in detail in Table 1).

As for the location of the lesions, seven were located in the stomach (five in gastric antrum, two in gastric body) and the other one in the lower esophagus. Among the four patients with GOV, the lesions were far from the GOV in three patients, while the other one patient had superficial esophageal cancer on the esophageal varices. Two patients received esophageal variceal ligation before endoscopic treatment, and the ESD was performed 2 and 4 weeks later, respectively. Two, three, and two patients received perioperative platelets, fresh frozen plasma, and albumin infusion, respectively.

### 3.2. Treatment Outcomes

ESD was performed successfully in all eight patients; the operation time ranged from 45 to 230 min. Intraoperative bleeding was encountered in four patients. For the patient with esophageal lesion, intraoperative bleeding owing to the submucosal esophageal varices and unintentional injury of the muscularis propria layer was encountered; therefore, a fully covered metal stent was inserted. And the stent may also help to prevent stricture that may result from circumferential ESD. The stent was removed 4 weeks later. The resected specimen measured from 2 × 1.8 cm to 6 × 2 cm, and en bloc resection was achieved in six of the eight patients (75%). As for pathological results, three were gastric adenocarcinoma, two were gastric high-grade intraepithelial neoplasia, one was gastric low-grade intraepithelial neoplasia, one was gastric adenoma, and the remaining one was esophageal squamous cell carcinoma (ESCC). Curative resection was achieved in five patients (62.5%). The patient who had ESCC had chest pain for 3 days and a fever for 4 days (with the highest temperature of 38.4 centigrade), and the symptoms were relieved with antibiotics. And postoperative pathological results revealed submucosal infiltration (sm2) with negative vertical and lateral margins. And further surgery was recommended; however, he refused. No recurrence or esophageal stricture was noticed during a follow-up of 5 months. For the seven patients with gastric lesions, no postoperative complication was reported, and no recurrence was noticed during a follow-up of 3 to 45 months (see in detail in Table 2).

## 4. Discussion

Liver cirrhosis ranks as the 11th most common cause of death globally, and China alone accounts for approximately 11% of the deaths [11,24]. Patients with liver cirrhosis have an increased incidence of esophageal and gastric cancers due to the fact that liver cirrhosis and esophageal/gastric cancers share some common risk factors, such as alcohol intake, smoking, obesity, etc. [25,26]. An increased prevalence of gastric cancer (1.9-fold) and esophageal cancer (2.6-fold) was observed in patients with liver cirrhosis as compared to the general population [26,27]. Usually, periodic endoscopy is performed for surveillance of GOV; the high incidence suggests that more attention should be paid to screening early esophageal/gastric cancers in liver cirrhosis patients. In the present study, seven out of the eight patients were found to have cancerous or precancerous lesions during screening or surveillance of GOV. However, little is known about the incidence of early esophageal/gastric cancer in patients with liver cirrhosis. Zhu et al. [14] reported that 4.24% (25/565) of patients had comorbid liver cirrhosis in patients with early esophageal cancers who underwent endoscopic submucosal tunnel dissection. In the present study, 0.94% of patients had comorbid liver cirrhosis among patients with early esophageal cancer/precancerous lesions who received ESD. We reported a proportion of 2.05% of patients who had liver cirrhosis in patients who received ESD for early gastric cancers or precancerous lesions. As far as we know, this is the first study that reported the incidence of comorbid liver cirrhosis in patients who received gastric ESD for early cancers or precancerous lesions.

Endoscopic resection has been recommended as the standard method for the treatment of early-stage esophageal/gastric cancers and precancerous lesions. ESD offers a higher rate of complete resection and en bloc resection and a lower rate of local recurrence than EMR; thus, it is recommended as the first choice, especially for lesions > 2 cm [9,10]. However, ESD in patients with liver cirrhosis is challenging due to the presence of GOV, ascites, poor tolerance of anesthesia, coagulation disorders, and/or thrombocytopenia. A systematic review and meta-analysis revealed that the pooled rates of immediate and delayed bleeding, perforation, and death during EMR and/or ESD in patients with cirrhosis were 9.5%, 6.6%, 2.1%, and 0.6%, with only an immediate bleeding rate higher than that of the general population [28]. A multicenter observational study that enrolled 134 early esophageal tumorous lesions in 112 patients with liver cirrhosis reported 3 (2.7%) perforations, 8 (7.1%) delayed bleedings, 8 (7.1%) instances of sepsis, 6 (5.4%) cirrhosis decompensations within 30 days, and 22 (19.6%) esophageal strictures [17]. A systematic review enrolling 68 gastric ESD patients reported a total of eight (13.1%) instances of post-ESD bleeding and one (1.6%) perforation [29]. As for intraoperative bleeding, Zhu et al. [14] reported a rate of 48% (12/25) during endoscopic submucosal tunnel dissection for early esophageal carcinoma in patients with liver cirrhosis, and Kwon et al. [30] reported a proportion of 47% during endoscopic resection of gastric mucosal lesions in patients with chronic renal failure and liver cirrhosis. In the present study, intraoperative bleeding was found in 50% (4/8) of patients, which is similar to the above reports.

To ensure the safety of invasive procedures such as ESD, it is important to properly manage coagulopathy and thrombocytopenia preoperatively. Usually, fresh frozen plasma or platelet infusion is recommended [13,23]. Repici et al. [29] found that INR > 1.33 and/or platelet count < 105,000/mm^3^ should be regarded as having an increased risk of bleeding following ESD. Meanwhile, some studies have suggested that INR < 1.5 and platelet count > 50,000/mm^3^ can be used as critical values for patients with cirrhosis in assessing tolerance of invasive surgeries [12,31]. In vitro studies have shown that a platelet count of (20–30) × 1000/mm^3^ is enough to produce thrombin for the maximum amplitude in thromboelastography to be normal [32]. Zhang et al. [21] reported a case with a platelet count < 30 × 1000/mm^3^, which increased to >30 × 1000/mm^3^ after infusion with frozen platelets before endoscopic treatment, and no obvious bleeding was observed during or after ESD in this case. In the present study, two of three patients with INR > 1.33 and both the two patients with platelet counts of <50,000/mm^3^ encountered intraoperative bleeding, which illustrated that platelet count of >50 × 1000/mm^3^ is desirable for endoscopic treatments in patients with liver cirrhosis.

Another major concern during ESD in liver cirrhosis patients is the presence of GOV, as it may increase the rate of bleeding and other complications, especially when the lesion is on or adjacent to GOV. Therefore, it is necessary to comprehensively assess the positional relationship between the lesions and GOV, the risk of variceal bleeding, GOV injury during the ESD procedure and infiltration or metastasis of delayed treatment of mucosal lesions, the endoscopist’s experience, and so on [21]. In our opinion, if the early cancer or precancerous lesion is far from the GOV, and patients have a low risk of variceal bleeding, ESD can be performed first; for patients with a high risk of variceal bleeding, ESD should be performed after management of GOV or simultaneously with management of GOV. No consensus has been reached regarding the optimal interval between GOV management and ESD in this situation; we suggest ESD be performed within 4 weeks of GOV management. In our study, three patients had upper gastrointestinal lesions far from GOV, and GOV was managed first in two of them, and ESD was performed 2 and 4 weeks later, and ESD was immediately performed in the other one patient as only mild esophageal varice was noticed. If the lesion is located overlying or adjacent to GOV, the risk of bleeding is increased remarkably. If a high risk of GOV exists, and the lesion has a low risk of infiltration or metastasis, GOV should be performed first; for patients with high-risk lesions and a low risk of variceal bleeding, ESD for the lesion should be performed first; for patients with both high risk of lesion and variceal bleeding, they could be managed simultaneously. In our study, one patient had esophageal cancer overlying the esophageal varices, ESD was successfully performed although intraoperative bleeding was encountered. As for the management strategy of GOV, no consensus was reached. Either endoscopic or interventional therapy is acceptable for patients with early cancer or precancerous lesions far from GOV. Some researchers recommend against endoscopic treatment as it may induce submucosal fibrosis which increases the technical difficulty of subsequent ESD [33,34]; therefore, transjugular intrahepatic portosystemic shunt (TIPS) or balloon-occluded retrograde transvenous obliteration was used to prevent variceal bleeding [17,35,36,37]. Endoscopic radiofrequency ablation and laparoscopic–endoscopic cooperative surgery have also been reported for the management of such patients [38,39]. Zhang et al. [21] reported the outcomes of ESD in 15 cases (had 16 lesions) with both GOV and upper gastrointestinal early cancer or precancerous lesions. Among the 16 mucosal lesions, 2 (12.5%) were located overlying the GOV, 6 (37.5%) were located beside the GOV, and 8 (50%) were located far from the GOV. And five (33.3%) were untreated for GOV during the ESD perioperative period, one (6.7%) was treated for GOV after ESD, six were treated for GOV before ESD, one (6.7%) was treated for GOV before ESD and during ESD, and two (13.3%) were treated for GOV during ESD. In a multicenter study, 134 lesions in 112 patients were treated using the endoscopic method, with esophageal varices in 71 procedures. To prevent bleeding, 7 patients underwent TIPS, 8 had EBL before resection, 15 received vasoactive drugs, and 9 underwent EBL during the resection procedure [17].

The present study has several limitations. Firstly, this was a single-center, retrospective study conducted in a tertiary hospital with a relatively small sample size. This may be due to the low incidence of such patients. A sample size of eight is not enough to draw a confirmed statement regarding the safety of ESD in these populations. Therefore, a large-scale study is warranted. Secondly, ESDs were all performed by experienced endoscopists in the present study, thus the results may not be directly generalized. Thirdly, no comparison with non-cirrhotic patients was made due to the small sample size. Fourthly, assessment of the liver function is necessary before ESD treatment; patients with Child–Pugh B/C or a history of hepatocellular carcinoma may benefit less from ESD [15,16]. Due to the limited sample size, we could not explore the relationship between Child–Pugh scores and treatment outcomes. Lastly, a recent meta-analysis revealed that liver cirrhosis was associated with a higher rate of recurrence in the long term [40]. No recurrence was noticed in the present study, possibly due to the small sample size and relative short-term follow-ups. Therefore, a large-scale study with a long-term follow-up is warranted.

## 5. Conclusions

In a word, our preliminary study revealed that ESD may serve as a safe and effective method for treating upper gastrointestinal early-stage cancers or precancerous lesions in patients with liver cirrhosis. Proper perioperative management of coagulation dysfunction, thrombocytopenia, GOV, and hypoalbuminemia is helpful in guaranteeing the safety of ESD.

## Figures and Tables

**Table 1:** Basic clinical characteristics of the 8 patients.

Case No.	Sex	Age (Year-Old)	Course of Liver Cirrhosis	Etiology of Liver Cirrhosis	Presence of GOV	History of GOV Therapy	PLT (×10^9^/L)	Hb (g/L)	INR	ALB (g/L)	Child–Pugh Score (Stadium)
1	Female	68	1 year	HBV	Yes	No	72	110	1.37	32.6	6 (A)
2	Female	70	5 years	cryptogenic	Yes	2 times	46	69	1.17	28.3	6 (A)
3	Female	59	1 week	HBV	No	No	35	115	1.18	36.1	5 (A)
4	Male	52	5 years	cryptogenic	Yes	No	96	113	1.43	27.5	9 (B)
5	Male	64	1 year	Alcoholic	Yes	3 times	56	111	1.45	32.3	8 (B)
6	Female	56	6 months	Budd-Chiari	No	No	81	147	0.93	44.5	5 (A)
7	Female	64	0	cryptogenic	No	No	169	99	1.03	32.8	7 (B)
8	Male	43	8 months	HCV	No	No	145	158	0.98	42.2	5 (A)

Abbreviations: GOV: gastroesophageal varices; PLT: platelet; Hb: hemoglobin; INR: international normalized ratio; ALB: albumin; HBV: hepatitis B virus; HCV: hepatitis C virus.

**Table 2:** Characteristics and treatment outcomes of the 8 patients.

Case No.	Location	Size (cm)	Relationship with GOV	Perioperative PLT Infusion	Perioperative Plasma Infusion	Operation Time (min)	Pathology	En Bloc Resection	Intraoperative Bleeding	Postoperative Complication	Follow-Up (Months)	Recurrence
1	Gastric body	2 × 2	Far from	No	No	110	HGIN	Yes	No	No	45	No
2	Gastric antrum	4 × 2	Far from	Yes	Yes	105	Tub1 m2	Yes	Yes	No	15	No
3	Gastric antrum	4.4 × 3.5	NA	Yes	No	70	Tub2 m3	Yes	Yes	No	3	No
4	Esophagus	6 × 2	On	No	Yes	180	ESCC sm2	Yes	Yes	Chest pain Fever	5	No
5	Gastric antrum	4.5 × 3.5	Far from	No	Yes	230	Tub1/Tub 2, m3	No	Yes	No	15	No
6	Gastric antrum	3 × 3	NA	No	No	70	LGIN	Yes	No	No	18	No
7	Gastric body	2 × 1.8	NA	No	No	45	Adenoma	Yes	No	No	15	No
8	Gastric antrum	2.6 × 2	NA	No	No	70	HGIN	No	No	No	16	No

Abbreviations: GOV: gastroesophageal varices; PLT: platelet; HGIN: high-grade intraepithelial neoplasia; ESCC: esophageal squamous cell carcinoma; NA: not available; LGIN: low-grade intraepithelial neoplasia.

## Data Availability

The data presented in this study are available upon request from the corresponding author.
